# HIV low-level persistent viremia under new antiretroviral regimens: what we have learned up to this point?

**DOI:** 10.1186/1471-2334-14-S7-O2

**Published:** 2014-10-15

**Authors:** Valeriu Gheorghiță, Loredana Benea, Alina Elena Barbu, Flavius Anghel, Ruxandra Moroti, Dragoş Florea, Otilia Elisabeta Benea, Florin Alexandru Căruntu

**Affiliations:** 1 Central Universitary Emergency Military Hospital Dr Carol Davila, Bucharest, Romania; 2 National Institute for Infectious Diseases "Prof. Dr. Matei Balş", Bucharest, Romania; 3 Carol Davila University of Medicine and Pharmacy, Bucharest, Romania

## Background

The current goal of antiretroviral treatment (ART) is to achieve and maintain virological suppression below limits of detection (<50 copies/mL). Despite a potent ART, some patients experience persistently low viral loads (VL), between 50-1000 copies/mL. The long-term consequences of persistent low-level viremia (LLV) are negative, usually predicting a virological failure.

## Methods

A cohort, retrospective study was conducted in Adult Clinic I of the National Institute for Infectious Diseases "Prof. Dr. Matei Balş", Bucharest, over a 6 year-period (01.2008-12.2013). The main inclusion criteria were: HIV-positive patients stable on ART (>6 months) at their first regimen, good adherence and absence of other medication susceptible for drug-drug interactions with ART.

We recorded the demographical data (age/gender), the HIV transmission route, CDC stage, the baseline immune-virological status (CD4 count, HIV-VL and genotypic mutations) and ART regimen. There were also registered subsequently CD4 count and HIV-VL (biannually taken). We then analyzed the management of the patients found with LLV: maintaining the current ART regimen and close monitoring, ART intensification or ART switch. LLV was defined as VL >50 and <1000 copies/mL in at least 2 determinations over a 24 week-period, after at least 24 weeks of stable ART.

## Results

Of 61 patients screened, 35 met the inclusion criteria. The median age was 38 years, (IQR, 32-51) and 71.4% (n=25) were male. According to 1993 CDC classification, 42.8% (n=15) were A2 and 22.8% (n=8) were C3. Sixty percent (n=21) were heterosexually infected. The median baseline CD4 count was 292 cells/cmm (IQR, 179-376), and median VL was 5.1 log_10_ copies/mL (IQR, 4.4-5.4). Two patients had detectable baseline mutations. All ART regimens contained 2 NRTI plus one as following: boosted-lopinavir (11 patients), efavirenz (10 patients), boosted-darunavir (6 patients), boosted-atazanavir (5 patients) and raltegravir (3 patients). Of 11 patients (31.4%) who had detectable VL at 6 months, 5 met the LLV definition criteria. Their median VL was 267 copies/mL and the median CD4 count was 551/cmm. None of them had had baseline mutations. They didn’t have changes in the current ART regimen, except one patient in whom we increased darunavir dose to 1200 mg/day. At 12 months their median CD4 count raised to 743/cmm and the median VL declined to 150 copies/mL.

## Conclusion

Although none of the patients with LLV became undetectable at 12 months of treatment, their VL levels decreased progressively in line with the increase in CD4 count in the absence of ART changing.

